# HDAC inhibitors as anticancer drugs: chemical diversity, clinical trials, challenges and perspectives

**DOI:** 10.1039/d5ra09034b

**Published:** 2026-01-15

**Authors:** Abdallah E. Abdallah

**Affiliations:** a Pharmaceutical Medicinal Chemistry & Drug Design Department, Faculty of Pharmacy (Boys), Al-Azhar University Cairo 11884 Egypt abdulla_emara@azhar.edu.eg

## Abstract

In an attempt to collect clinical data about HDAC inhibitors as very significant anticancer drugs we aimed to compare data and reveal the impact of the structural features, concluding the points of interest that are likely to help further development of better cancer therapy. We presented results of different clinical phases of HDAC inhibitors classified as hydroxamic acid derivatives, cyclic peptides, benzamides, and short chain aliphatic acids in a coherent and cohesive manner. It was found that HDAC inhibitors are preferentially combined with other antitumor drugs, mainly anti PD-1 and doxorubicin. In contrast, drugs such as docetaxel exaggerate the toxicity of HDAC inhibitors. Furthermore, data from clinical trials showed that the efficacy of HDAC inhibitors as single agents was limited against solid tumors. But they were significant against many solid tumors when combined with other anticancer agents. For example, combination of vorinostat and doxorubicin showed good results in solid tumors, especially prostate cancer, breast cancer, and melanoma. On the contrary, single agents of HDAC inhibitors revealed considerable clinical outcomes against different types of lymphoma and leukemia that warrant further investigation. Meanwhile, combinations of HDAC inhibitors and other drugs were also effective against lymphomas and leukemias.

## Abstract 2

In an attempt to collect clinical data about HDAC inhibitors as very significant anticancer drugs, we aimed to compare data and reveal the impact of the structural features to help – develop better cancer therapy.

## Graphical abstract

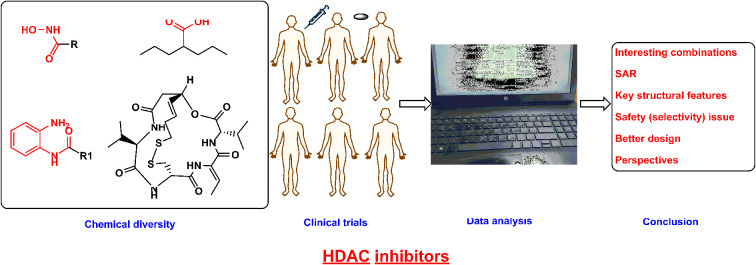

## 1. Introduction

Cancer is one of the most challenging diseases that threaten people worldwide.^1^ The diversity of related cellular processes reveals a very considerable challenge to thoroughly repair cellular defects attributed to cancer. No single target can be identified for treatment of all cancers. Acquired resistance to cancer therapy is a major issue.^2^ Moreover, the clinical uses of some chemotherapeutic agents are likely to show non tolerated adverse effects.^3^ Many factors contribute to the difficulty of cancer therapy, keeping it as a disease of high mortality rate all over the world.^4,5^ On the other side, there has been a significant improvement in treatment of cancer. A high level of understanding of cellular defects specific to cancer cells uncovered the most crucial targets for cancer treatment. Great efforts have been made in discovering and testing many targeted molecules in preclinical and clinical trials. More effort may be required for collecting data concerning specific targets for analyzing and suggesting or taking more effective decisions. Herein, we attempted to structure collected comprehensive data of histone deacetylase (HDAC) (*e.g.* characteristics, pharmacophores of related molecules, preclinical and clinical results), aiming at concluding some inspiring perspectives for development of better inhibitors. Additionally, we aimed at drawing attention to some gaps in the literature. The current work analyzes the structural features of the HDAC inhibitors in relation to the clinical data, compares structural classes chemically and biologically, collects interesting combinations, and reports the recent clinical studies.

### 1.1. Function and types of HDAC

Initially, HDACs are detected in almost all tissues as key enzymes for histone deacetylation.^6^ Histone is not the sole substrate of HDAC but the most important in the sense that it is an essential component of chromatin that is composed of DNA wrapping around histone protein.^6,7^ Post-translational modification of histone by acetylation or deacetylation of NH of its conserved lysine residue is balanced by histone acetyl transferase (HAT) and HDAC.^8^ Histone acetylation as a covalent modification of histone does not lead to alteration in DNA sequences but epigenetic changes, controlling the rate of transcription and gene expression.^9,10^ Eighteen isoforms of HDACs have been identified and classified into three main categories, I, II, and III, on the basis of structural characterization.^11,12^ Class I includes HDACs 1, 2, 3, and 8. They were proven to play a crucial role in transcriptional repression, differentiation inhibition, and cell cycle progression.^7,12–14^ The functions of HDAC 1, 2, and 3 are achieved through binding to large proteins, forming multiprotein complexes known as NuRD, CoREST, and Sin3, which are recruited to chromatin 14. Class II HDAC comprises six members: HDACs 4, 5, 7, 9 (class IIa), and HDACs 6 and 10 (class IIb). Their cellular functions are related to regulation of transcription, cell differentiation, migration and inflammation.^14–16^ HDAC class I and class II are Zn^2+^ dependent enzymes, in contrast to class III enzymes that are NAD^+^ dependent.^11,17,18^ Class III members are SIRT1-7 and their functions are linked to metabolism, stress response, aging, and cell cycle.^14,19^ HDAC11 was considered as the sole member of a distinct class known as HDAC class IV.^20^ It was found to be Zn^2+^ dependent and highly associated with obesity, tumor growth, and prognosis.^21,22^

It was evident that HDAC is a significant target for discovery of potent anticancer drugs.^23–25^ The role of HDAC in tumor growth may be attributed to post-translational regulation of essential angiogenesis factors; hypoxia inducible factor (HIF-1 α) and vascular endothelial growth factor (VEGF).^14,26^ As mentioned above, different isoforms of HDAC are linked to cell cycle progression, differentiation inhibition, and tumor growth. There is some evidence that inhibition of HDAC reduces angiogenesis and induces cell cycle arrest, mitotic cell death, and autophagic cell death.^27–31^ In addition, HDAC inhibitors enhanced apoptosis with high selectivity to cancer cells.^32,33^ The association of HDAC with tumor was further proven by reporting overexpression of HDAC in some tumor types.^34,35^ For example, HDAC1 and HDAC2 were reported to be overexpressed in breast cancer and colon cancer, respectively.^34,36–38^ Four distinct chemical classes of HDAC inhibitors are defined, we will discuss them in this review considering FDA approved drugs and those that are in clinical trials. These chemical categories are hydroxamic acid derivatives, cyclic peptides, benzamides, and short chain aliphatic acids.^13^

## 2. Chemical classes of HDAC inhibitors

### 2.1. Hydroxamic acid derivatives

**Fig. 1:**
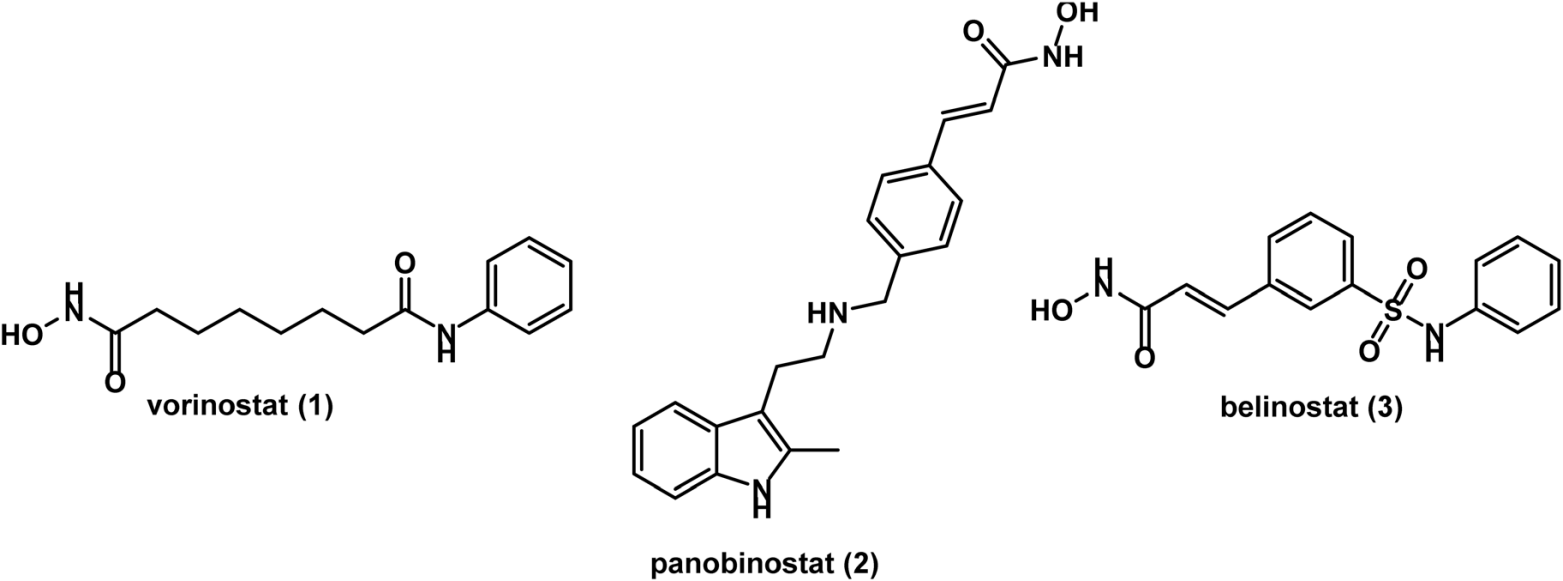
Illustration of chemical structures of FDA approved hydroxamic acid derivatives.

This class showed inhibition of enzymes of HDAC classes I, II, and IV.^13,26^ The first HDAC inhibitor approved by FDA for cancer treatment was vorinostat or suberoylanilide hydroxamic acid (SAHA) 1. It was approved for cutaneous T-cell lymphoma (CTCL).^39,40^ Further two hydroxamic acid derivatives; panobinostat 2 and belinostat 3 (identified by Novartis), have been approved for multiple myeloma (MM) and peripheral T-cell lymphoma (PTCL), respectively^41^ (see Fig. 1). The FDA approval in 2015 for panobinostat was accelerated as it was indicated for life threatening MM, but FDA withdrew this approval in 2022, and hence panobinostat is no longer indicated for MM.^42^

In 2008, a phase II study indicated the limited efficacy and high toxicity of vorinostat 1 in patients with recurrent and metastatic transitional cell urothelial cancer.^43^ Conversely, the combination of vorinostat and doxorubicin was found to be effective with good tolerability in patients with prostate cancer, breast cancer, and melanoma, as a phase I study reported in 2009.^44^ While a phase I study of a combination of vorinostat and docetaxel in patients with solid tumors was early terminated due to excessive toxicity.^45^ In 2019, a phase I trial indicated the safety and high efficacy of the combination of vorinostat and chemoradiation therapy for treatment of head and neck squamous cell carcinoma.^46^

In 2012, a phase II study revealed that panobinostat as a monotherapy showed antitumor activity with durable response and manageable adverse events in patients with relapsed and refractory Hodgkin's lymphoma.^47^ In 2014, a phase I study suggested that panobinostat plus erlotinib is a well-tolerated and effective double therapy regimen in patients with non-small cell lung cancer (NSCLC) and those with head and neck cancer.^48^ In 2016, a phase II clinical trial showed that panobinostat 2 induces durable responses, showing a 28% response rate in patients with diffuse large B-cell lymphoma (DLBCL).^49^ Furthermore, the addition of rituximab did not improve the effect of panobinostat in patients with DLBCL.^49^

In 2016, a combination of belinostat and doxorubicin was evaluated in a phase I/II study conducted on patients with soft tissue sarcomas. The results indicated the well tolerability of this combination with some improvement in progression time compared to doxorubicin alone, however, there was no evidence of synergy between belinostat and doxorubicin in soft tissue sarcomas.^50^ In 2021, a phase I study evaluated belinostat and bortezomib combination in relapsed or refractory acute leukemia and myelodysplastic syndrome revealed insignificant overall activity. However, some exceptional responses observed to this combination warrant further investigation.^51^

**Fig. 2:**
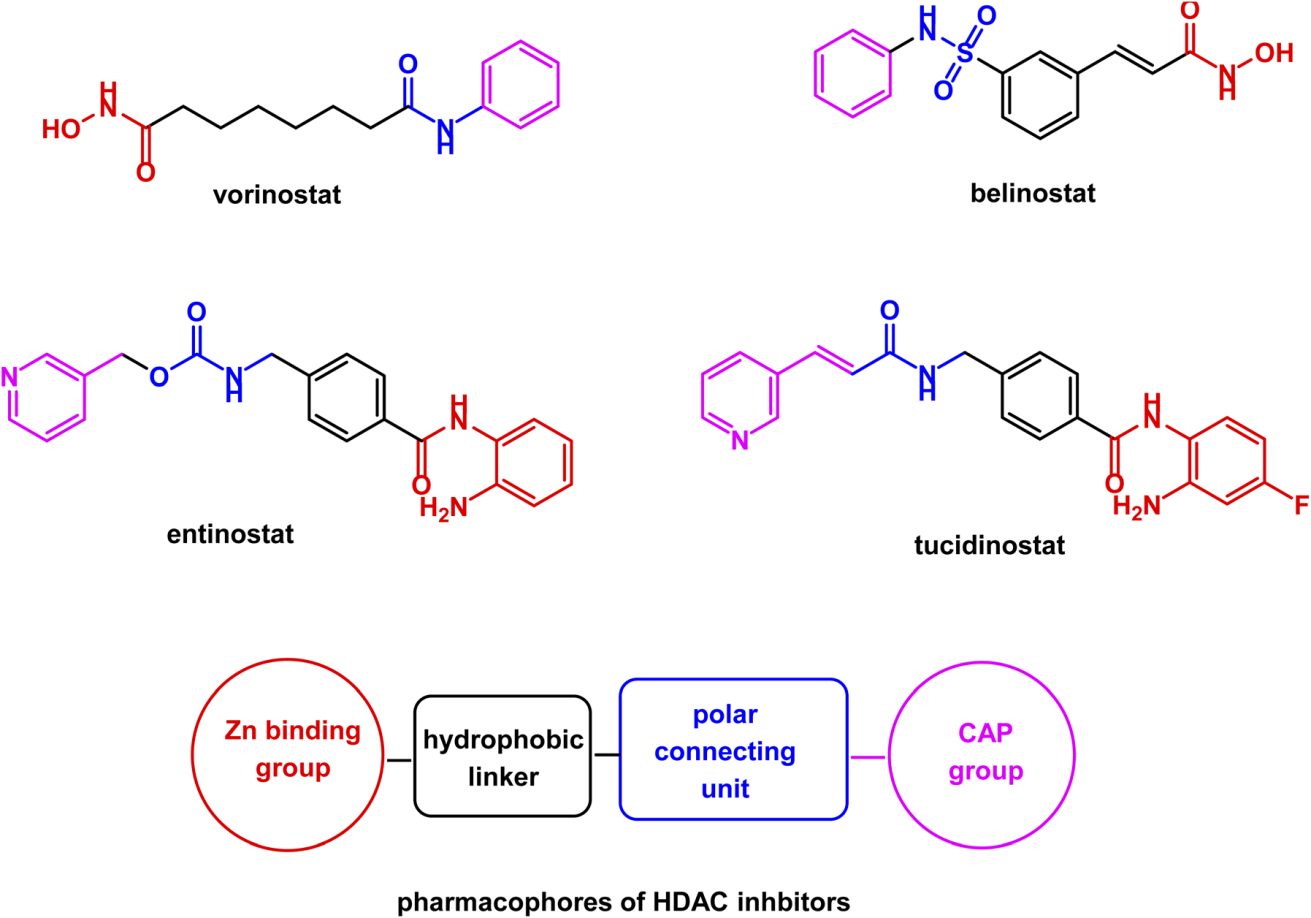
Illustration of HDAC inhibitors pharmacophoric features common for hydroxamic acid derivatives and benzamides.

The common structural features of these drugs can be noticed from a glance at their chemical structures. They include a hydroxamic acid group (a Zn binding group) linked through a lipophilic spacer to a polar group attached to a terminal hydrophobic aromatic ring, representing a surface recognition part^52^ (see Fig. 2).

**Fig. 3:**
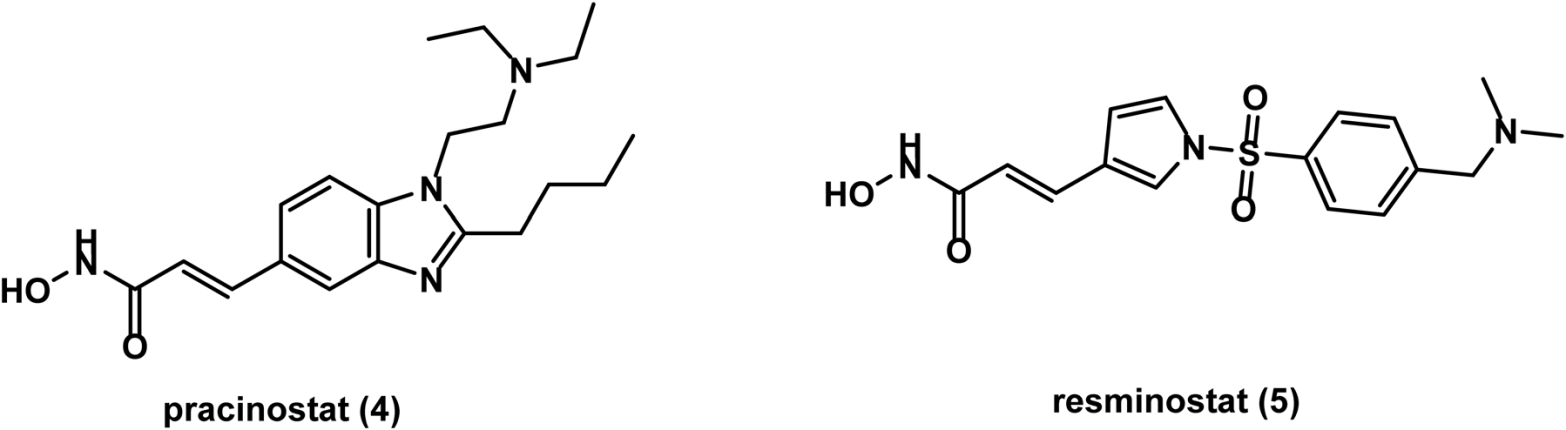
Chemical structures of the hydroxamic acid derivatives, pracinostat and resminostat.

A phase 1 study conducted on pracinostat (SB939) 4 (Fig. 3) in 2011 reported that it is tolerable in patients with advanced solid tumors and shows side effects consistent with those of other HDAC inhibitors.^53^ Another phase I study in 2013 showed that it is well tolerated in children with refractory solid tumors.^54^ A phase II study of pracinostat conducted in 2015 on patients with castration resistant prostate cancer (CRPC) revealed no sufficient activity to warrant further study, however, it was well tolerated and showed a decline in circulating tumor cells (CTC).^55^

In 2013, a phase I study of resminostat (4SC-201) 5 (Fig. 3) as an oral HDAC inhibitor revealed good safety in patients with advanced solid tumors.^56^ In 2021, data of a phase II study on resminostat indicated neither improvement in progression free survival nor overall survival in patients with pretreated biliary tract cancer (BTC).^57^

**Fig. 4:**
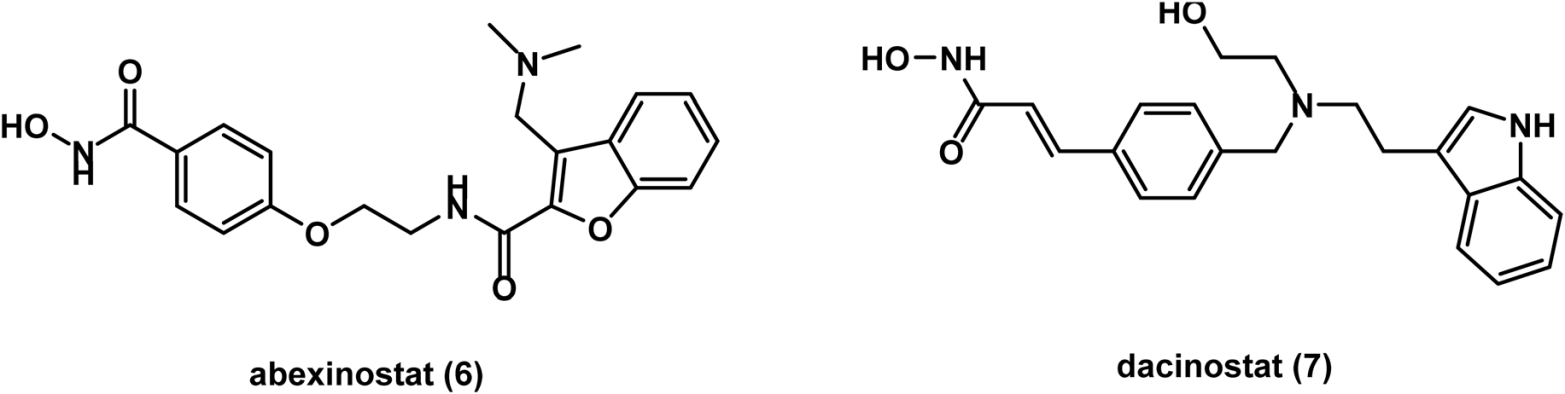
Chemical structures of the hydroxamic acid derivatives abexinostat and dacinostat.

In 2008, abexinostat (S78454/PCI-24781) 6 (Fig. 4) was evaluated in a phase I study, which reported that abexinostat is orally bioavailable and is well tolerated when administered IV.^58^ In 2016, a phase I/II study on patients with relapsed/refractory lymphoma revealed high tolerability with significant activity that warrant further trials.^59^ A subsequent phase II study showed high activity and favorable tolerability of abexinostat in patients with relapsed/refractory non-Hodgkin lymphoma (NHL).^60^ A recent phase I study confirmed the high safety of abexinostat in chinese patients with relapsed/refractory β-cell NHL.^61^

Dacinostat (NVP-LAQ-824) 7 (Fig. 4), which discovered by Novartis, was found to be well tolerated when administered I.V. in a phase I study conducted on patients with advanced solid tumors in 2008.^62^ However, phase II studies on dacinostat have been terminated due to a toxicity issue.^63^

**Fig. 5:**
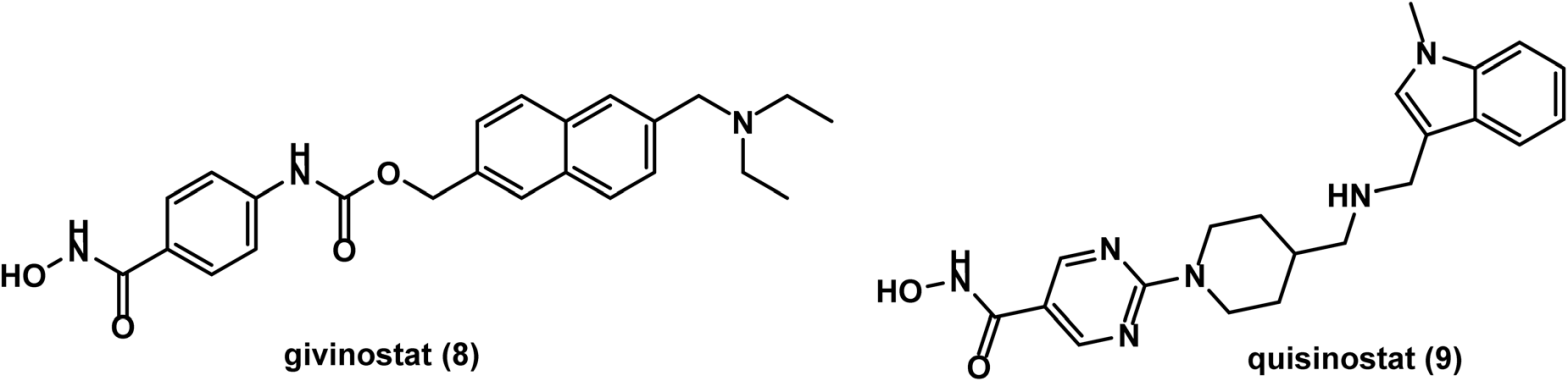
Chemical structures of the hydroxamic acid derivatives, givinostat and quisinostat.

Givinostat (ITF2357) 8 (Fig. 5), discovered by Italfarmaco, was found to be tolerable and an inhibitor to pro-inflammatory cytokine production without affecting anti-inflammatory cytokines in a phase I study conducted in 2011.^64^ In 2020, a phase 1B/II study suggested givinostat as a well tolerated and promising therapy in polycythemia vera.^65^ In 2023, data of a phase II study showed that givinostat failed to prevent or delay the progression of Becker muscular dystrophy (BMD), however, MRI assessment may be considered a potential signal suggesting givinostat could slow down BMD progression.^66^ A recent phase III trial indicated that the efficacy of givinostat in Duchenne muscular dystrophy (DMD) was beyond corticosteroids. However, further investigations into long term safety and cost-effectiveness are still required.^67^

Quisinostat (JNJ-26481585) 9 (Fig. 5), a second generation hydroxamic acid derivative with specific subnanomolar activity against class I of HDAC, especially HDAC 1 and 2.^68^ In 2012, quisinostat was found to be effective with a good safety profile in treatment of CTCL according to results of a phase II study designed for evaluation of oral quisinostat in previously treated CTCL.^69^ In 2013, the clinical results showed that quisinostat had good tolerability and significant antitumor activity against advanced solid tumors, especially melanoma.^70^ This was confirmed in 2016 by a subsequent phase II study that reported favorable safety and efficacy for quisinostat in treatment of patients with relapsed or refractory CTCL.^71^ Further investigation in a phase II trial concluded promising efficacy and safety of quisinostat in combination with paclitaxel and carboplatin in treatment of patients with recurrent platinum resistant ovarian cancer.^72^

**Fig. 6:**
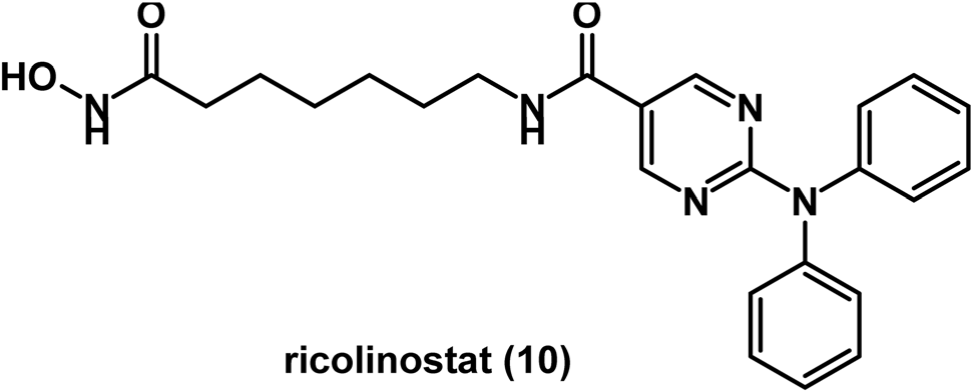
Chemical structure of the hydroxamic acid derivative, ricolinostat.

Ricolinostat (ACY-1215) 10 (Fig. 6) was developed as a selective HDAC 6 inhibitor in order to avoid several adverse effects recorded for pan-HDACi. Data of preclinical investigation in MM model showed synergy between ricolinostat and bortezomib. Preliminary results of phase I/II trials suggested that ricolinostat is likely to be a well-tolerated treatment for relapsed or refractory MM either alone or in combination with other drugs such as bortezomib and dexamethasone.^73^ This was confirmed by results of the first part of a phase Ib study that reported that ricolinostat is well tolerated in combination with bortezomib and dexamethasone in patients with relapsed or refractory MM.^74^ In 2021, data of a phase Ib/II study indicated that oral ricolinostat was safe and stabilized half of the evaluated patients with relapsed and refractory lymphoma.^75^

### 2.2. Cyclic peptides

**Fig. 7:**
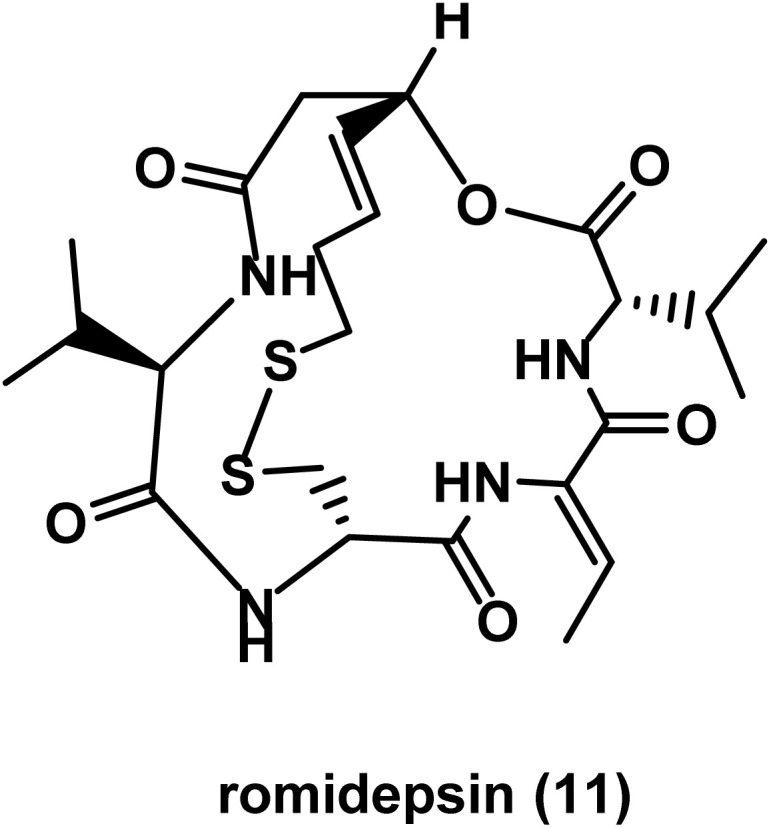
Chemical structure of the cyclic peptide romidepsin.

The natural cyclodepsipeptide, romidepsin 11 (Fig. 7), was approved for CTCL and PTCL.^76,77^ It is considered a broad spectrum HDAC inhibitor, acting mainly on class I HDAC and at relatively higher concentrations on class II HDAC.^78^ In 2024, data of a phase 1b/2a trial revealed the significance of a combination of a phosphoinositide-3-kinase inhibitor such as duvelisib and a HDAC inhibitor such as romidepsin in T-cell lymphoma.^79^

### 2.3. Benzamides

**Fig. 8:**
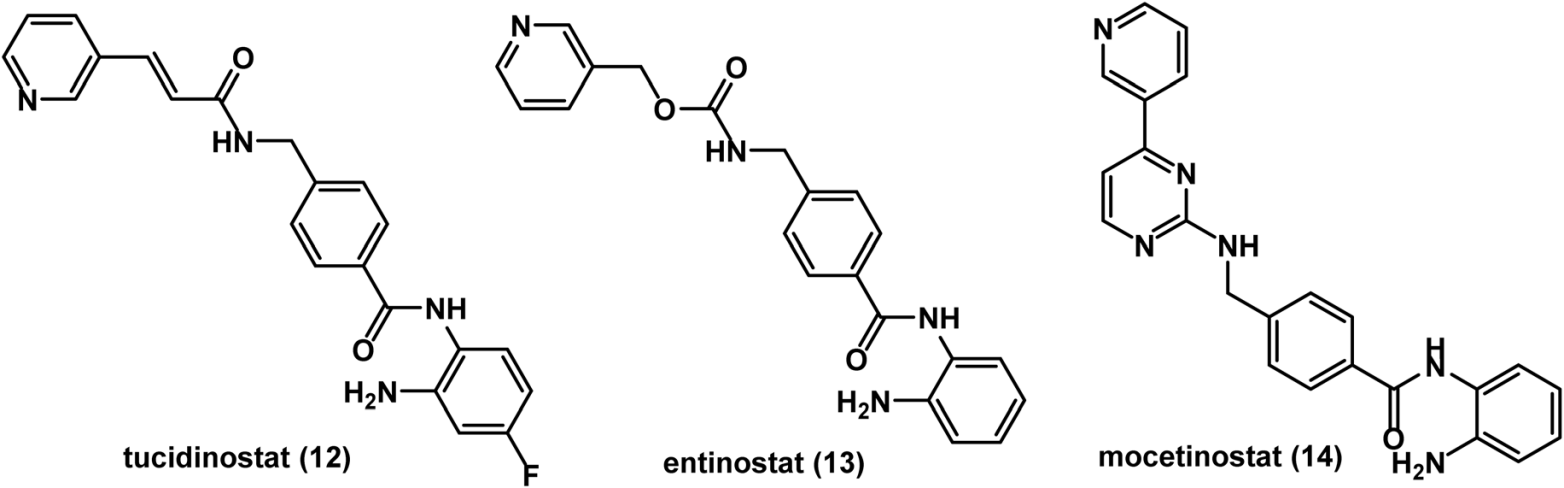
Chemical structures of the approved benzamide derivatives, tucidinostat, entinostat, and mocetinostat.

One of the most interesting HDAC inhibitors is benzamides class in the sense that its activity is almost specific to class I HDAC, which is highly related to tumor growth and metastasis. As a consequence, selective HDAC inhibitors are more likely to be effective as anticancer agents with fewer adverse effects than other pan-HDAC inhibitors. Tucidinostat (chidamide) 12, entinostat 13, and mocetinostat 14 are examples of clinically used anticancer benzamides^80,81^ (see Fig. 8).

Tucidinostat 12 did not receive approval from FDA, but it was approved by Chinese authority for PTCL and advanced breast cancer.^82–84^ Meanwhile, FDA granted entinostat 13 a breakthrough therapy designation for advanced breast cancer.^85^ While mocetinostat 14 received from FDA an orphan drug designation for DLBCL.^86,87^ Benzamides have common pharmacophoric features similar to those of hydroxamic acid derivatives, including a Zn binding group, a hydrophobic spacer, a polar connection unit, and a CAP group.^88,89^ Clinical trials of pan-HDAC inhibitors documented a wide range of side effects.^90^ So, benzamides as selective HDAC class 1 inhibitors may be preferred for drug development.

Several clinical trials have been conducted on benzamide derivatives as HDAC inhibitors, while others are in progress. In 2021, results obtained from a phase II study revealed that tucidinostat 12 as a monotherapy induced durable tumor regression with a 25% overall response rate and a 15% overall response in patients with relapsed or refractory DLBCL.^91^ A clinical study published in 2025 indicated that tucidinostat 12 improved clinical outcomes in adult patients with relapsed or refractory T-cell leukemia (ATL), showing a 54.2% objective response rate (ORR) and a 91.7% disease control rate (DCR).^92^ A similar earlier phase IIb study reported a 30.4% ORR, 7.9 months as median overall survival (OS), and 1.7 months as median progression free survival (PFS), suggesting the significance of tucidinostat as a treatment option for relapsed or refractory ATL. However, all patients experienced adverse effects that were mainly hematologic and gastrointestinal.^93^ A phase III trial examined the combination of tucidinostat and the steroidal aromatase inhibitor (AI) exemestane in comparison to exemestane alone for treatment of hormonal receptor (HR) positive breast cancer in postmenopausal patients. The study revealed that the combined therapy caused a significant improvement in PFS with no change in OS.^94^ It was also reported that 51.6% of patients in the tucidinostat group experienced neutropenia of grade 3 or 4 compared to 2.5% of placebo group patients.^94^ In 2022, clinical results showed that mPFR was 4.5 months in patients with HR positive metastatic breast cancer treated with tucidinostat sequentially after a prior CDK4/6 inhibitor.^95^ So the sequential combination of tucidinostat and endocrine therapy is likely to be an effective approach for treatment of patients with HR positive advanced breast cancer.^95^ Data obtained from a phase II study, conducted in the years 2021 and 2022, showed that a combination of tucidinostat and tislelizumab (an anti-programmed death receptor-1 (PD-1) antibody) is a very potent regimen, with a good safety profile, for treatment of locally advanced or metastatic urothelial carcinoma. It showed a 41.7% ORR, a 62.5% DCR, a median PFS of 4.6 months, and adverse effects of grade 1 or 2.^96^ In 2024, data of a phase II study suggested that tucidinostat plus pediatric chemotherapy is a potent and tolerated regimen for patients with early T-cell precursor lymphoblastic leukemia/lymphoma (ETP-ALL/LBL), showing high negative rates of composite complete remission (CCR) and minimal residual disease (MRD) as well as promising survival outcomes. Meanwhile, most of the patients experienced grade 3–4 adverse effects such as neutropenia, anemia, and thrombocytopenia.^97^ In 2022, a phase I study concluded that tucidinostat showed promising efficacy and safety with mild to moderate hematological toxicities in patients with non-Hodgkin lymphoma.^98^ In 2024, a phase II study revealed the encouraging efficacy and acceptable safety of tucidinostat plus toripalimab (an anti PD-1 antibody) in patients with metastatic or unresectable melanoma.^99^ In 2025, the combination of tucidinostat, bortezomib, liposomal doxorubicin, and dexamethasone was clinically proven to be an effective regimen in relapsed and refractory multiple myeloma.^100^

In 2021, the results of a phase III trial enrolled on men and women suggested that a combination of entinostat 13 and exemestane does not improve survival of patients with AI resistant, advanced HR positive, human epidermal growth factor-2 (HER-2) negative breast cancer.^101^ A phase II study for evaluation of the combination of entinostat and nivolumab as a PD-1 inhibitor on patients with advanced pancreatic ductal adenocarcinoma (PDA) showed no significance with respect to ORR. However, durable responses were observed in a small subset of patients.^102^ Another phase II trial did not show a clinical efficacy for the combination of entinostat and nivolumab in cholangiocarcinoma.^103^ In 2021, data of a phase I study revealed that entinostat is highly tolerable in children with relapsed or refractory solid tumors, showing good pharmacokinetics and pharmacodynamics that encourage further evaluation in phase II trials.^104^

As a single agent, mocetinostat 14 showed limited efficacy in patients with relapsed and refractory chronic lymphocytic leukemia, according to data of a phase II study.^105^ It was also evaluated as a single agent in a phase II study involved patients with relapsed classical Hodgkin's lymphoma. The data obtained indicated promising activity with a bad safety profile due to dose limiting toxicity.^106^ Similarly, in a phase II study, mocetinostat as a monotherapy failed to treat patients with urothelial carcinoma due to a toxicity issue.^107^ In 2023, a phase I/II study reported that the combination of mocetinostat and durvalumab (an anti PD-1 antibody) showed a durable response (median 329 days) with a good safety profile in patients with NSCLC unresponsive to prior anti PD-1 therapy.^108^ On the other side, according to results of a phase I/II trial, the combination of mocetinostat and gemcitabine showed high toxicity and limited clinical outcomes in patients with advanced pancreatic cancer.^109^ In two separate phase Ib studies, despite the favorable response rates, the toxicity was high for the triple regimen of mocetinostat, ipilimumab, and nivolumab in patients with unresectable or metastatic melanoma.^110,111^ A phase I study concluded that mocetinostat did not improve objective responses when it was used in combination with 13-*cis* retinoic acid in patients with solid tumors. However, longer durations of stable disease were observed in patients with kidney, prostate, and pancreatic cancers.^112^

**Fig. 9:**
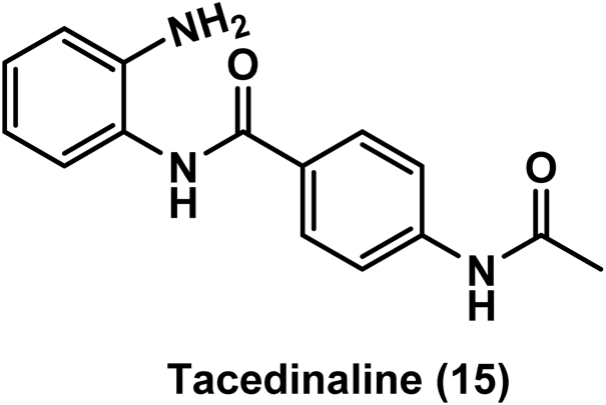
Chemical structure of the benzamide, tacedinaline.

Tacedinaline (CI-994) 15, a relatively simple benzamide derivative as shown in Fig. 9, caused growth inhibition at both the main site and metastatic regions in two orthotopic mouth models of MYC driven medulloblastoma, according to data of a clinical study.^113,114^

### 2.4. Short chain aliphatic acids

**Fig. 10:**
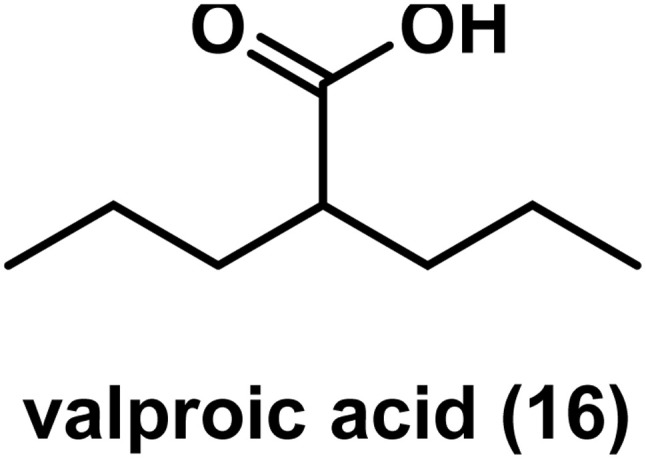
Chemical structure of valproic acid.

Valproic acid 16 (Fig. 10) is a short chain aliphatic acid with HDAC inhibition. In 2007, according to a phase I/II study, the combination of valproic acid, 5-azacitadine and all *trans* retinoic acid revealed 42% ORR with good tolerability in patients with acute myeloid leukemia (AML) or high risk myelodysplastic syndrome (MDS). In untreated older patients, ORR was found to be 52%.^115^ It also showed an improvement as a monotherapy in 44% of patients with AML and MDS, according to data of a phase 1 study.^116,117^ In 2011, data of a small phase II study reported that valproic acid activates Notch1 signaling and decreases apoptosis markers, and hence it is likely to play a role in treating neuroendocrine carcinoma.^118^ In 2016, a phase II study indicated the effectiveness of cisplatin, cetuximab, and valproic acid as a well-tolerated first-line triple chemotherapy regimen in patients with metastatic and recurrent squamous cell carcinoma of the head and neck.^119^

## 3. Conclusion

We summarized the clinical trials conducted on HDAC inhibitors, in Tables 1–4, in a manner that shows the drugs examined against a specific cancer type collected with their results in order to easily compare and reach a more valuable conclusion. Furthermore, the current work considers some issues in the current clinical research, such as failure due to toxicity. The study attempted to get insights into the chemical features related to activity and/or toxicity in order to help researchers develop more effective and safe derivatives.

**Table 1:** Solid tumors involved in clinical trials of different HDAC inhibitors as a monotherapy as well as the results obtained

Serial	Cancer type	Study phase	Therapy regimen	Results
1	Solid tumors	Phase I	Quisinostat	Good tolerability and significant antitumor activity especially against melanoma^70^
	Solid tumors	Phase 1	Pracinostat	Tolerable even in children^53,54^
	Solid tumors	Phase I	Resminostat	Good tolerability^56^
	Solid tumors	Phase I	Dacinostat	Well tolerated when administered I.V.^62^
	Solid tumors	Phase II	Dacinostat	Terminated due to a toxicity issue^63^
	Solid tumors	Phase I	Entinostat	Highly tolerable in children^104^
2	Castration resistant prostate cancer	Phase II	Pracinostat	No significant activity, but it was well tolerated^55^
3	Recurrent and metastatic urothelial cancer	Phase II	Vorinostat	Limited efficacy and high toxicity^43^
	Recurrent and metastatic urothelial cancer	Phase II	Mocetinostat	Failed due to a toxicity issue^107^
4	Pretreated biliary tract cancer	Phase II	Resminostat	No improvement in clinical outcomes^57^

**Table 2:** Lymphomas and leukemias involved in clinical trials of different HDAC inhibitors as a monotherapy as well as the results obtained

Serial	Cancer type	Study phase	Therapy regimen	Results
1	Hodgkin s lymphoma	Phase II	Panobinostat	Showed some activity with good tolerability^47^
	Hodgkin's lymphoma	Phase II	Mocetinostat	Promising activity with bad safety profile^106^
2	Relapsed/refractory non-Hodgkin lymphoma	Phase II	Abexinostat	High activity and favorable tolerability^60^
	Non-Hodgkin lymphoma	Phase I	Tucidinostat	Promising efficacy and safety^98^
3	Diffuse large B-cell lymphoma	Phase II	Panobinostat	28% Response rate^49^
	Diffuse large B-cell lymphoma	Phase II	Tucidinostat	25% Overall response rate and a 15% overall response^91^
4	Relapsed or refractory lymphoma	Phase I/II	Abexinostat	High tolerability with significant activity^59^
	Relapsed or refractory lymphoma	Phase Ib/II	Ricolinostat	Safe and stabilized half of the evaluated patients of^75^
5	Relapsed or refractory T-cell leukemia	Phase II	Tucidinostat	Significant improvement in clinical outcomes^92^
	Relapsed or refractory T-cell leukemia	Phase IIb	Tucidinostat	Significant improvement in clinical outcomes^93^
6	Cutaneous T-cell lymphoma	Phase II	Quisinostat	Effective with good safety profile^69^
	Cutaneous T-cell lymphoma	Phase II	Quisinostat	Favorable safety and efficacy^71^
7	Chronic lymphocytic leukemia	Phase II	Mocetinostat	Limited efficacy^105^
8	Acute myeloid leukemia and myelodysplastic syndrome	Phase 1	Valproic acid	An improvement in 44% of patients^116,117^

**Table 3:** Solid tumors involved in clinical trials of different HDAC inhibitors in a combination as well as the results obtained

Serial	Cancer type	Study phase	Therapy regimen	Results
1	Breast cancer	Phase I	Vorinostat plus doxorubicin	Effective and well tolerated^44^
2	HR positive breast cancer	Phase III	Tucidinostat plus exemestane	Improvement in PFS with no change in OS. Neutropenia was a serious adverse event^94^
	HR positive breast cancer	Phase II	Tucidinostat sequentially after a prior CDK4/6 inhibitor	Effective sequential approach with mPFR of 4.5 months^95^
3	AI resistant, HR^+^, HER-2^−^ breast cancer	Phase III	Entinostat plus exemestane	No improvement in survival of patients^101^
4	Solid tumors	Phase I	Mocetinostat plus 13-*cis* retinoic acid	No improvement in objective responses^112^
	Solid tumors	Phase I	Vorinostat plus docetaxel	Early terminated due to excessive toxicity^45^
	Solid tumors	Phase I	Vorinostat plus doxorubicin	Effective and well tolerated in particular against prostate cancer, breast cancer and melanoma^44^
5	Metastatic or unresectable melanoma	Phase II	Tucidinostat plus toripalimab	Promising efficacy and good safety^99^
	Metastatic or unresectable melanoma	Phase Ib	Mocetinostat, ipilimumab and nivolumab	Good response rates, but toxicity was high^110,111^
6	Non-small cell lung cancer	Phase I/II study	Mocetinostat and durvalumab	Durable response (median 329 days) with good safety profile^108^
	Non-small cell lung cancer	Phase I	Panobinostat plus erlotinib	Well-tolerated and effective double therapy regimen^48^
7	Head and neck cancer	Phase I	Panobinostat plus erlotinib	Well-tolerated and effective double therapy regimen^48^
8	Metastatic and recurrent squamous cell carcinoma of the head and neck	Phase II	Cisplatin, cetuximab plus valproic acid	Effective regiment with good tolerability^119^
9	Recurrent and metastatic urothelial cancer	Phase II	Tucidinostat plus tislelizumab	Potent efficacy with good safety profile^96^
10	Advanced pancreatic cancer	Phase I/II	Mocetinostat plus gemcitabine	High toxicity and limited clinical outcomes^109^
11	Advanced pancreatic ductal adenocarcinoma	Phase II	Entinostat plus nivolumab	No significant clinical results^102^
12	Cholangiocarcinoma	Phase II	Entinostat plus nivolumab	No significant clinical efficacy^103^
13	Recurrent platinum resistant ovarian cancer	Phase II	Quisinostat, paclitaxel and carboplatin	Promising efficacy and safety^72^
14	Soft tissue sarcomas	Phase I/II	Belinostat and doxorubicin	Well tolerability with some improvement progression time^50^

**Table 4:** Liquid tumors involved in clinical trials of different HDAC inhibitors in a combination as well as the results obtained

Serial	Cancer type	Study phase	Therapy regimen	Results
1	Multiple myeloma	Phase I	Tucidinostat, bortezomib, liposomal doxorubicin, and dexamethasone	Effective clinical results
	Multiple myeloma	Phase Ib	Ricolinostat plus bortezomib and dexamethasone	Well tolerated combination^74^
2	T-cell lymphoma	Phase 1b/2a	Romidepsin plus duvelisib	Significant clinical outcomes^79^
3	T-cell precursor lymphoblastic leukemia/lymphoma	Phase II	Tucidinostat plus pediatric chemotherapy	Potent and tolerated regimen^97^
4	Relapsed or refractory acute leukemia and myelodysplastic syndrome	Phase I	Belinostat plus bortezomib	Insignificant overall activity
5	Acute myeloid leukemia and myelodysplastic syndrome	Phase I/II	Valproic acid, 5-azacitadine and all *trans* retinoic acid	Good clinical outcomes with favorable tolerability^115^

The hydroxamic acid is the most effective chemical class against lymphomas and MM. Consequently, three hydroxamates, which are vorinostat, panobinostat, and belinostat received FDA approval for CTCL, MM, and PTCL, respectively. On the contrary, no benzamide derivative has received FDA approval for lymphoma yet.

We can notice that all chemical classes of HDAC inhibitors act well with good safety when combined with some other drugs against different cancer types, as illustrated in Tables 3 and 4. As can be seen, vorinostat plus doxorubicin showed good results in solid tumors, in particular prostate cancer, breast cancer, and melanoma. Tucidinostat and toripalimab combination was found to be potent against melanoma. The double therapy regimen of panobinostat plus erlotinib was effective against NSCLC and head and neck cancers. While the combination of cisplatin, cetuximab, and valproic acid was promising in squamous cell carcinoma of head and neck. Meanwhile, tucidinostat plus tislelizumab revealed efficacy against urothelial carcinoma. Whereas in ovarian cancer, quisinostat, paclitaxel, and carboplatin combination was effective. We can also notice that tucidinostat, when combined with bortezomib, liposomal doxorubicin, and dexamethasone, showed good results in MM. Also romidepsin plus duvelisib combination was significant in T-cell lymphoma. Meanwhile, tucidinostat plus pediatric chemotherapy showed potent results against T-cell precursor lymphoblastic leukemia/lymphoma. Additionally, valproic acid, 5-azacitadine, and all *trans* retinoic acid regimen was promising in acute myeloid leukemia and myelodysplastic syndrome. On the other side, some combinations of HDAC inhibitors, including hydroxamic acid or benzamide derivatives, failed due to a toxicity issue such as tucidinostat plus exemestane, vorinostat plus docetaxel, mocetinostat, ipilimumab, and nivolumab combination, and mocetinostat plus gemcitabine. Furthermore, other combinations showed a limited efficacy, such as entinostat plus exemestane in AI resistant, HR positive, HER-2 negative breast cancer. Similarly, mocetinostat plus 13-*cis* retinoic acid in solid tumors and belinostat plus bortezomib in relapsed or refractory acute leukemia. Likewise, entinostat plus nivolumab in both advanced pancreatic ductal adenocarcinoma and cholangiocarcinoma.

Regarding clinical studies on HDAC inhibitors as a monotherapy, both hydroxamic acid and benzamide derivatives showed good tolerability against solid tumors as outlined in Table 1. But the hydroxamic acid derivatives vorinostat and dacinostat and the benzamide mocetinostat displayed high toxicity in solid tumor studies. On the other side, as listed in Table 2, many hydroxamic acid derivatives showed significant activity and tolerability against lymphomas and leukemias. Meanwhile, tucidinostat was the only benzamide candidate that revealed considerable data against lymphomas and leukemias. We can see in Table 1 that in solid tumor phase I trials, quisinostat, pracinostat, resminostat, and entinostat were tolerable. Whereas, dacinostat was toxic in phase II trials against solid tumors. In castration resistant prostate cancer, pracinostat was not effective, however, tolerability. In recurrent and metastatic urothelial cancer, vorinostat showed toxicity and limited efficacy. Similarly, mocetinostat failed due to toxicity. In pretreated biliary tract cancer, resminostat showed no improvement. Table 2 reveals that in Hodgkin's lymphoma, panobinostat showed good results, while mocetinostat had a bad safety profile. Additionally, in non-Hodgkin's lymphoma, both abexinostat and tucidinostat were effective. Furthermore, in DLBCL, both panobinostat and tucidinostat showed good results. Also, in relapsed or refractory lymphoma, abexinostat and ricolinostat were significant. In relapsed or refractory T-cell leukemia, tucidinostat was effective. Similarly, in CTCL, quisinostat was promising. And finally, in acute myeloid leukemia and myelodysplastic syndrome, valproic acid showed some good results.

## 4. Expert opinion

Upon analyzing the results of clinical studies of different chemical structures of hydroxamic acid derivatives, it can be noticed that presence of a hydrogen bond donor atom in the polar group attached to the lipophilic linker is a key feature for activity. This feature is presented in many drugs such as vorinostat, belinostat, and entinostat. It was found that molecules that lack the hydrogen bond donor atoms showed weak activities, as can be noticed from the results of pracinostat and resminostat. Furthermore, it can be noticed that branching on the spacer of the hydroxamic acid derivatives is likely to increase toxicity, as was reported in dacinostat, which displayed dose limiting toxicity. Another aspect of safety profile is that selective HDAC inhibitors are more likely to be safer than pan-HDAC inhibitors. The concrete example of this point is that benzamides are safer than hydroxamic acid derivatives to the extent that entinostat was found to be safe for children with solid tumors. This fact is likely not to be attributed to selectivity only, but it may also be related to the stronger binding affinity of hydroxamic acid group to Zn than amide and amine groups, exaggerating the adverse effects associated with binding to other metalloproteases.

We can notice that the most serious adverse events reported for benzamide derivatives were hematologic side effects. These adverse effects were more predominant in tucidinostat, where neutropenia was very serious, to a level that led to discontinuation of some clinical studies. Meanwhile, tucidinostat was very effective in patients with lymphomas and leukemias. In comparison to other benzamides, the relatively high therapeutic and adverse effects of tucidinostat on blood cells may be attributed to the fluorine atom attached to anilide moiety. Further optimization of tucidinostat may be required to improve safety and hence be approved by FDA. Alternatively, it can be combined with other therapies to limit its toxicity as well as potentiate activity. The combination of tucidinostat and anti-PD-1 drugs was proven to be a highly effective and encouraging regimen for many cancer types. Mocetinostat failed as a single agent in different clinical trials due to a toxicity issue. The incorporation of guanidino group instead of amide (as in tucidinostat) or carbamate (as in entinostat) may account for the bad safety profile. With respect to hydroxamic acid derivatives, they can be combined with doxorubicin to afford good synergistic effects with better safety, especially against solid tumors.

One of the key structural differences between hydroxamic acid derivatives and benzamides is the Zn binding group. It is stronger, more flexible, and terminal with less steric hindrance in hydroxamic acid derivatives. These features collectively potentiate the binding to Zn and broaden the activity of hydroxamic acid to include different HDAC isoforms. It was reported that HDAC classes I (HDACs 1, 2, 3, and 8), II (HDACs 4, 5, 6, 7, 9, and 10), and IV (HDAC 11) are inhibited by hydroxamic acid derivatives. They are all Zn dependent HDACs and play roles in tumor growth. This may account for their toxicity reported clinically and it may explain why hydroxamic acid derivatives were more active against lymphomas and leukemias than benzamide derivatives. It was proven that benzamides are almost selective HDAC class I inhibitors. However, both classes share the pharmacophores essential for HDAC inhibition, giving comparable data in many clinical trials as discussed above.

## Conflicts of interest

There is no conflict of interest and this work was funded by the author.

## Data Availability

The data supporting this article have been included in the references.
